# Case report: Familial glycogen storage disease type IV caused by novel compound heterozygous mutations in a glycogen branching enzyme 1 gene

**DOI:** 10.3389/fgene.2022.1033944

**Published:** 2022-11-08

**Authors:** Yiyang Li, Chuan Tian, Si Huang, Weijie Zhang, Qiuyu Liutang, Yajun Wang, Guoda Ma, Riling Chen

**Affiliations:** ^1^ Department of Pediatrics, Shunde Women and Children’s Hospital of Guangdong Medical University, Foshan, China; ^2^ Department of Pediatrics, Affiliated Hospital of Guangdong Medical University, Zhanjiang, China; ^3^ Department of Pediatrics, The Third Affiliated Hospital of Guangzhou Medical University, Guangzhou, China; ^4^ Key Laboratory of Research in Maternal and Child Medicine and Birth Defects, Guangdong Medical University, Foshan, China

**Keywords:** GSD IV, GBE1, asymptomatic hypoglycemia, liver transplantation, case report

## Abstract

Glycogen storage disease type IV (GSD IV), caused by a mutation in the glycogen branching enzyme 1 (GBE1) gene, is a rare metabolic disorder with an autosomal recessive inheritance that involves the liver, neuromuscular, and cardiac systems. Here, we reported a case of familial GSD IV induced by novel compound heterozygous mutations in GBE1. The proband (at age 1) and her younger brother (at age 10 months) manifested hepatosplenomegaly, liver dysfunction, and growth retardation at onset, followed by progressive disease deterioration to liver cirrhosis with liver failure. During the disease course, the proband presented rare intractable asymptomatic hypoglycemia. The liver pathology was in line with GSD IV. Both cases carried pathogenic compound heterozygous mutations in GBE1 mutations, i.e., a missense mutation (c.271T>A, *p*. W91R) in exon 2 and a deletion mutation in partial exons 3–7. Both mutations are first reported. The internationally pioneered split-liver transplantation was performed during progression to end-stage liver disease, and the patients had normal liver function and blood glucose after. This study broadens the mutation spectrum of the GBE1 gene and the phenotypic spectrum of GSD IV.

## Introduction

Glycogen storage disease type IV (GSD IV, OMIM 232500, Anderson disease) is a rare metabolic disorder with autosomal recessive inheritance. It is caused by a mutation in the glycogen branching enzyme 1 (GBE1) gene, which encodes the glycogen branching enzyme that catalyzes glycogen synthesis, with an incidence rate of 1/600,000–1/800,000 (Magoulas et al., 1993; Choi et al., 2018; Massese et al., 2022).

GBE1 catalyzes α-1,6-glucosidic bonds of the glycogen molecule, transferring the oligosaccharide chain containing six glucose residues to the adjacent fourth glucose molecule to form branches, thereby increasing glycogen solubility. Mutations in the GBE1 gene can lead to decreased or missed GBE1 activity, resulting in the accumulation of immature amylopectin-like polysaccharides in tissues (including the liver, skeletal muscle, cardiac muscle, brain, and peripheral neuropathy) (Magoulas et al., 1993; Li et al., 2010). The severity of the phenotype might depend on the residual activity of GBE1 (Massese et al., 2022).

With approval by the Medical Ethics Committee of the hospital and obtaining informed consent from the proband’s parents, this study reported a case of familial GSD IV caused by compound heterozygous mutations in GBE1. The proband had rare intractable asymptomatic hypoglycemia.

## Case presentation

**FIGURE 1:**
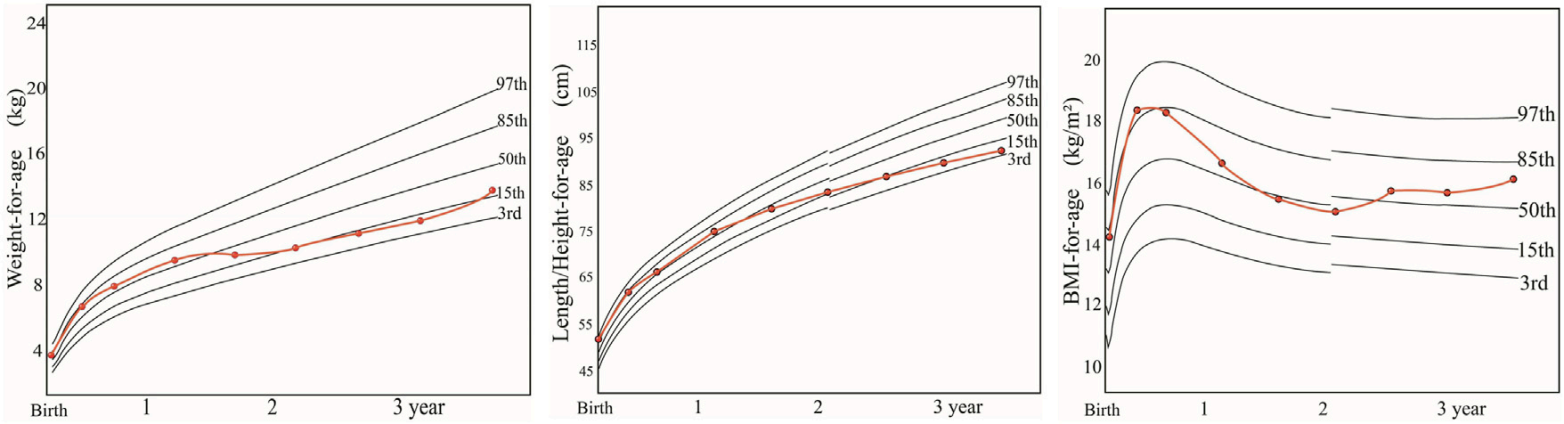
Growth and development of the proband within 3 years after birth. Note: weight and body mass index (BMI): normal nutrition and growth before the age of 1 year; moderate nutrition and insufficient weight gain after the age of 1 year; improved nutrition and growth after liver transplantation. Length/height: normal length/height and growth before the age of 1 year; inadequate growth after the age of 1 year; no improvement after liver transplantation, showing growth retardation. By now, the proband has a symmetrical short stature.

The female proband was diagnosed with malnutrition and growth retardation at age 1 on physical examination (Figure 1), accompanied by moderate hepatosplenomegaly with a hard texture, transaminase elevation, and progressive liver function deterioration on routine physical examination.

**FIGURE 2:**
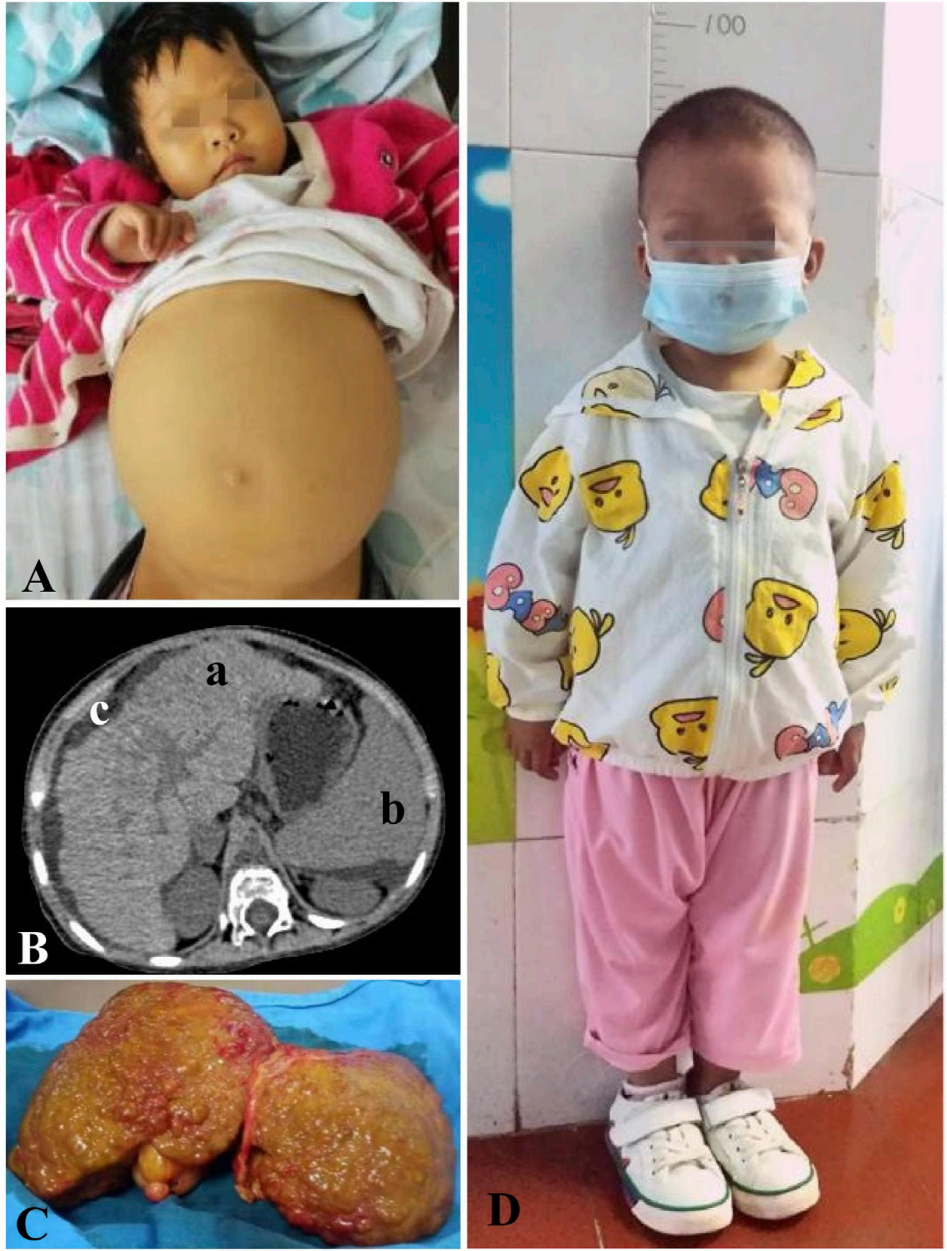
Clinical data of the proband. **(A)** End-stage liver disease, accompanying distension over the whole abdomen and severe yellowing of the skin. **(B)** Upper abdominal plain CT scan: (a). enlarged liver with a coarse, nodular texture and serrated capsule. (b). Enlarged spleen. (c). Ascites. **(C)** Lesion liver, an enlarged liver with a diffuse, gray–green coarsely granular appearance to the surface. **(D)** Proband 6 months after the transplantation, aged 3 years and 7 months, showing a symmetrical short stature with height 93 cm (3^rd^–15th) and weight 13.8 kg (15^th^–50th).

At the age of 2.5 years, she manifested distension over the whole abdomen (Figure 2A) with severe yellowing of the skin and sclera and umber urine and without any symptoms of hypoglycemia. On physical examination, her height, weight, and head circumference were 86 cm, 11 kg, and 46 cm, respectively (Figure 1). Moreover, she had malnutrition. Her liver extended 1 cm above the umbilicus, and her spleen extended 2 cm below the umbilicus. Both the liver and spleen had a hard texture. Shifting dullness was positive. Moreover, she presented symmetrical pitting edema in bilateral lower limbs.

### Test and treatment

Alanine aminotransferase (ALT), 133 U/L (9–50 U/L); aspartate transaminase (AST), 380U/L (9–48 U/L); AST/ALT, 2.8; glutamyl transpeptidase, 74 U/L (0.0–53.0 U/L); total bile acid, 202 μmol/L (0.0–15.0 μmol/L); total bilirubin, 139 μmol/L (2.0–20.0 μmol/L); direct bilirubin, 105 μmol/L (0.3–6.0 μmol/L); albumin, 22 g/L (38.0–54.0 g/L); prothrombin time 29 s (10.6–14.3 s); activated partial thromboplastin time, 65 s (26–40 s); fibrinogen, 0.9 g/L (2.0–4.0 g/L); serum ammonia, 173 μmol/L (16–72 μmol/L); dynamically-monitored fasting plasma glucose (FPG) < 3.9 μmol/L (3.9–6.0 μmol/L), minimum, 1.2 μmol/L; and blood lactate, 3.2 mmol/L (0.6–2.4 mmol/L). Blood acylcarnitine analysis: low concentrations of free carnitine and multiple acylcarnitines. Routine blood test: white blood cell, 5.5 × 10^9^/L (4.0–10.0 × 10^9^/L); hemoglobin, 76 g/L (110–130 g/L); and platelet, 49 × 10^9^/L (100–300 × 10^9^/L). Bone marrow biopsy demonstrated myeloproliferation. The upper abdominal plain CT scan showed liver cirrhosis (Figure 2B). Pathology revealed an enlarged liver with a diffuse, gray–green coarsely granular appearance to the surface (Figure 2C). By light microscopy, nodular liver cirrhosis (G4S4) with pseudolobules was demonstrated. The liver cells showed diffuse swelling with sedimented ground-glass inclusion bodies (IBs) and slightly eccentric nuclei. Histopathological investigation showed positive staining for periodic acid-Schiff (PAS). It also showed cholestasis, significant proliferation in interstitial fibrous tissues, and small bile ducts, with massive infiltration of lymphocytes.

The disease progressed to decompensated cirrhosis with liver failure (Child–Pugh class C). Coagulation function was improved by plasma supplementation. Albumin infusion and diuresis were obtained to alleviate edema. Meanwhile, the patient was on dietary management and blood glucose monitoring. At the age of 3 years and 1 month, the patient received split-liver transplantation, followed by standard immunosuppressive therapy. She was regularly followed-up postoperatively for 1.5 years until now, presenting normal aminotransferase, peripheral blood cells, blood glucose, and a symmetrical short stature (Figure 2D).

### Family history

The proband is a Chinese girl. The parents and older sisters did not have liver disease, and the parents were non-consanguineously married. Her younger brother presented hepatosplenomegaly with liver injury (ALT, 290 U/L; AST, 390 U/L) at the age of 10 months, and he experienced progression to liver cirrhosis at the age of 1 year and 5 months accompanied by growth retardation. Normal FPG was detected by multiple tests. Split-liver transplantation followed by standard immunosuppressive therapy was performed at the age of 1 year and 7 months, and he presented normal liver enzyme levels during the 1-year follow-up until now.

## Genetic testing and analysis

### Genetic testing

Peripheral blood samples (2 ml) were collected from the proband, her parents, and her younger brother. Whole-exome sequencing was performed to detect potential mutations. Sanger sequencing was used for point mutation validation. qPCR was used to determine copy number variation with the target sequences.

**FIGURE 3:**
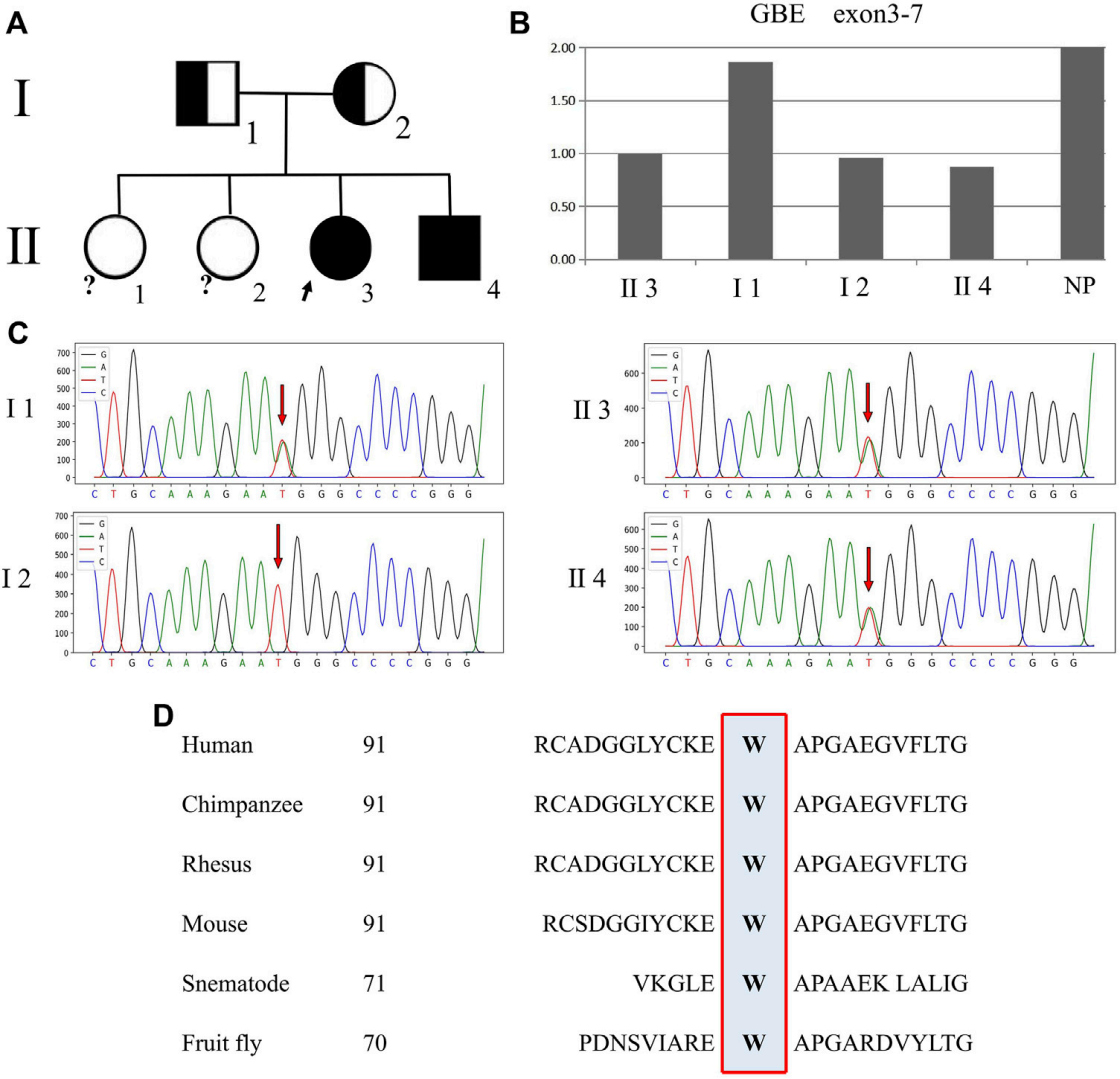
Genetic pedigree map and a GBE1 gene test result. Note: Ⅰ 1, father; Ⅰ 2, mother; Ⅱ 1, older sister 1; Ⅱ 2, older sister 2; Ⅱ 3, proband; and Ⅱ 4, younger brother; NP normal population. **(A)** Genetic pedigree map. The genotype of the proband’s two older sisters is unknown. **(B)** qPCR peak figure for the partial exons of the GBE1 gene. The relative quantitation values of exons 3–7 (chr3:81691916–81720106) in the GBE1 gene of the proband, mother, and younger brother are about 1/2 of the normal control value, suggesting a heterozygous deletion mutation. **(C)** Sanger sequencing. Arrows point to the missense mutation. 271T>A (*p*.W91R) in the GBE1 gene carried by the proband, her father, and her younger brother. The mother has a wild-type genetic structure. **(D)** Homology alignment for the amino acid sequences corresponding to the GBE1 missense mutation across different species.

Compound heterozygous mutations in the GBE1 gene were detected in the proband and her younger brother, including a heterozygous missense mutation (c.271T>A, *p*. W91R) in exon 2 (chr3: 81754637, genome version: hg19, transcript: NM_000158) inherited from the father and a heterozygous deletion mutation in partial exons 3–7 (chr3: 81691916–81720106, genome version: hg19, transcript: NM_000158:c.314_991del) inherited from the mother (Figure 3), which were compatible with recessive inheritance. Both parents are heterozygous carriers. Co-segregation between the phenotype and genotype in the proband and her family members was revealed.

### Variation in pathogenicity

The heterozygous missense mutation in the GBE1 gene, c.271T>A (*p*.W91R), is not included in multiple human variant databases (1000 Genomes Project (1000G) http://browser.1000genomes.org, ESP6500 http://evs.gs.washington.edu/EVS/, Exome Aggregation Consortium (ExAC) http://exac.broadinstitute.org/) and disease databases [Human Gene Mutation Database (HGMD)] http://www.hgmd.org, ClinVar http://www.ncbi.nlm.nih.gov/clinvar, and Genome Aggregation Database (gnomAD) http://gnomad.broadinstitute.org/about), and it is not a polymorphic mutation. With Clustal Omega software, this mutated locus encodes the amino acids which are highly conserved in evolution across humans, chimpanzees, rhesus, mice, nematodes, and fruit flies (Figure 3D). As predicted by online databases SIFT (http://sift.jcvi.org), PolyPhen2 (http://genetics.bwh.harvard.edu/pph2), MutationTaster (http://www.mutationtaster.org), and CADD (http://cadd.gs.washington.edu), the locus is located between conserved amino acid sequences, and mutation in this locus can lead to an altered RNA splice site, amino acid sequence, and eventually, protein structure, classified as a pathogenic mutation. Prediction by SAAFEC (http://compbio.clemson.edu/SAAFEC/userInputParams.html) indicates that mutation in this locus can cause protein-free energy change (ΔΔG = -1.61), suggesting decreased protein stability.

The exon 3–7 deletion in the GBE1 gene is not included in the human databases DGV (http://dgv.tcag.ca/dgv/app/home) and gnomAD-SV. Moreover, deletions in a smaller range are recorded in the HGMD database, including the pathogenic exon 2–7 deletion mutation (Bruno et al., 2004) and the pathogenic exon 7 deletion mutation (Li et al., 2012). The exon 3–7 deletion mutation results in a frameshift mutation, leading to a premature termination codon and the production of a truncated protein.

Based on the 2020 American College of Medical Genetics and Genomics (ACMG) criteria (Brandt et al., 2020; Riggs et al., 2020), the c.271T>A (in line with PM1, PM2, PP1, PP2, PP3, and PP4) and exon 3–7 deletions (in line with PVS1, PS1, and PM2) in the GBE1 gene are classified as pathogenic mutations.

### Analysis of the spatial structure of variant proteins

**FIGURE 4:**
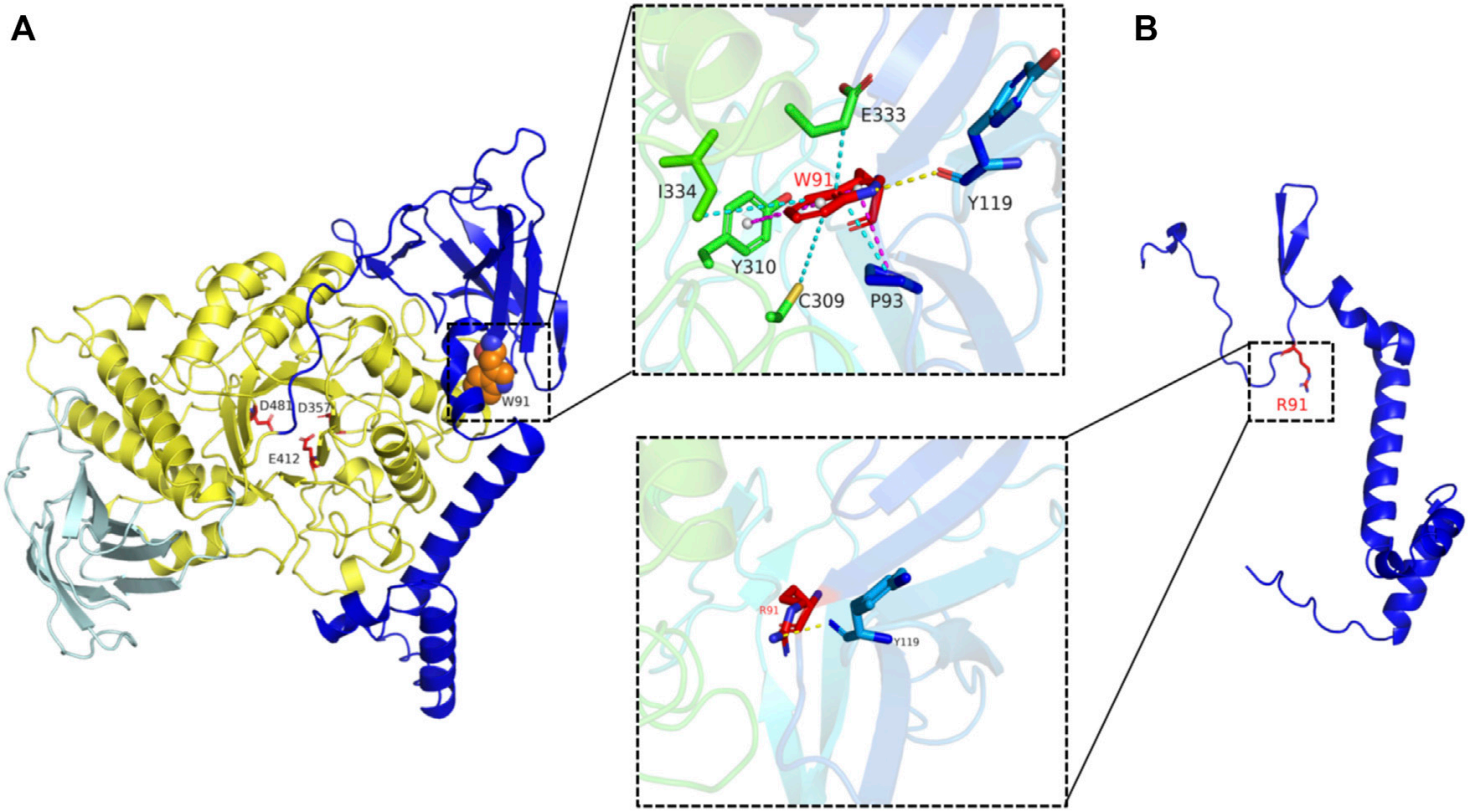
3D Wild-type structure and the mutated structure of GBE1 protein. Note: **(A)** Wild-type (WT) structure. The enzyme catalytic domain (amino acid sequence: 194–597) is colored yellow, and the three enzyme catalytic sites (D357, D481, and E412) are highlighted in red. W91 has hydrophobic interactions with E333, I334, Y310, C309, and P93 amino acids (blue dotted line), is hydrogen-bonded to the amino acid main chain of Y119 (yellow dotted line), and has π–π interactions with P93 amino acids (pink dotted line). **(B)** Mutated-type (MT) structure. W91R (panel below), all hydrophobic interactions and π–π interactions are lost, and only the hydrogen bonds with Y119 remain. Exon 3–7 deletion mutation, the catalytic domain at amino acid position 194–597 of GBE1 was lost.

Homology modeling of GBE1 was performed using AlphaFold 2 (https://alphafold.ebi.ac.uk), and the 3D structure of the protein was visualized using PyMOL 2.3 software (Figure 4A).

The tryptophan (Trp) residue at position 91 of GBE1 is located in the ligand-binding region and is highly conserved in the β-sheet secondary structure. The missense mutation c.271T>A changed Trp at position 91 to arginine (Arg), leading to local change from hydrophobic amino acids with aromatic side chains to strongly basic amino acids with positively charged side chains. As a consequence, the interactions between amino acid side chains of polypeptides and the electrostatic effects were altered, π–π interactions with P93 were lost, the hydrophobic interactions with amino acids E333, I334, Y310, C309, and P93 were lost, the locally hydrophilic property was enhanced, and the backbone was changed, leading to alteration in the spatial conformation of the protein (Figure 4B).

GBE1 is a glycogen-branching enzyme containing 702 amino acids. Exon 3–7 deletion in the GBE1 gene brought about deletion of the amino acid at position 105–331. After transcriptional splicing between a nucleotide at the end of exon 2 (the first nucleotide of *p*.105) and a nucleotide at the front of exon 8 (the last nucleotide of *p*.331), frameshift mutation presented in the amino acids from exon 8, and a shift of four amino acids resulted in a premature termination codon UAA, production of a truncated protein, and residual amino acid sequences at position 1–107. Eventually, the catalytic domain at amino acid position 194–597 of GBE1 was lost (Figure 4B).

## Discussion

GSD IV involves multiple systems, based on which it can be classified into hepatic and neuromuscular subtypes with significant clinical heterogeneity (Magoulas et al., 1993; Li et al., 2010). Pediatric cases are mainly hepatic subtypes. Its clinical manifestations include hepatomegaly, liver dysfunction, and progressive liver cirrhosis, potentially with neuromuscular lesions, cardiomyopathy, and growth retardation. Linear glycogen molecules can be metabolized, so often without hypoglycemia (Magoulas et al., 1993; Massese et al., 2022). The proband (at age 1) and her younger brother (at age 10 months) both manifested hepatosplenomegaly, dysfunction, and growth retardation at onset, followed by disease progression to liver cirrhosis with liver failure, consistent with the pathology of GSD IV. During the disease course, the proband presented rare intractable asymptomatic hypoglycemia which is easily neglectable. Since long-term hypoglycemia impacts the energy supply to the brain tissues and then impairs brain development and even causes sudden death, the blood glucose of patients with GSD IV should be dynamically monitored.

The diagnosis of GSD IV is established by the demonstration of reduced GBE1 activity in the liver, muscle, or skin fibroblasts and/or the identification of biallelic pathogenic variants in GBE1 (Magoulas et al., 1993; Ozen, 2007; Massese et al., 2022). Both the proband and her younger brother carried previously unreported compound heterozygous mutations in the GBE1 gene, including an exon 2 missense mutation (c.271T>A) and partial exon 3–7 deletion mutation. Homology modeling revealed that the missense mutation c.271T>A changed Trp at position 91 to Arg, leading to potential changes in the spatial conformation and property of the protein. Additionally, it may also affect the catalytic domain stability due to loss of hydrophobic interactions with amino acids E333, I334, Y310, and C309, eventually interfering with enzyme catalytic activity. The exon 3–7 deletion mutation in the GBE1 gene led to loss of the enzyme catalytic domain and then decline in catalytic activity.

Symptomatic treatment is the mainstay for treatment of GSD IV now, and there is no available enzyme replacement therapy (Massese et al., 2022). A strict dietary regimen includes a high protein diet and carbohydrate restriction. This can maintain glucose and lipid homeostasis, to minimize glycogen accumulation and catabolism; is fundamental to prevent hypoglycemia in ketotic GSD IV; and can even improve growth and normalize serum aminotransferases (Derks et al., 2021). Most of the patients with a progressive GSD IV would die from liver failure or other complications of liver cirrhosis within 5 years of age, and some may deteriorate to liver cancer that can only be cured by liver transplantation. However, liver transplantation cannot alleviate the glycogen accumulation in other organs and tissues in general (Magoulas et al., 1993; Beyzaei et al., 2022; Massese et al., 2022). Therefore, the extent of organ involvement (neuromuscle, heart, etc.) is the primary prognostic factor for liver transplantation, especially cardiomyopathy (Willot et al., 2010; Liu and Sun, 2021; Beyzaei et al., 2022). It should be noted that a previous report demonstrated improvement with significant reduction in abnormal amylopectin in extrahepatic organs in patients receiving liver transplantation (Ozen, 2007). The two subtypes of GSD IV, including the hepatic and neuromuscular subtypes, can occur successively (Derks et al., 2021). Previous literature reported a case where the patient presented with hepatic GSD IV at the onset at the age of 2 years and then had neuromuscular involvement at the age of 45 years (Paradas et al., 2014). Although no extrahepatic organ involvement has been observed in the two cases here so far, the existing literature reported a case of neuromuscular involvement caused by deletion in a range smaller than exon 3–7 (Bruno et al., 2004; Li et al., 2012). A follow-up is required to detect extrahepatic involvement as they grow up, including electrocardiogram, echocardiography, neurologic assessment, and nutritional assessment.

In conclusion, the clinical phenotype and genotype of GSD IV are highly heterogeneous. Most patients have poor prognosis, and patients with suspected GSD IV should be aggressively managed by liver biopsy and test for GBE activity or GBE1 gene mutation to obtain a definite diagnosis.

## Data Availability

The datasets for this article are not publicly available due to concerns regarding participant/patient anonymity. Requests to access the datasets should be directed to the corresponding author.

## Abbreviations

GSD IV, glycogen storage disease type IV; GBE1, glycogen branching enzyme 1; and PAS, periodic acid-Schiff.
